# Parental Influence on Child and Adolescent Physical Activity Level: A Meta-Analysis

**DOI:** 10.3390/ijerph192416861

**Published:** 2022-12-15

**Authors:** Diana L. Y. Su, Tracy C. W. Tang, Joan S. K. Chung, Alfred S. Y. Lee, Catherine M. Capio, Derwin K. C. Chan

**Affiliations:** 1 Department of Early Childhood Education, The Education University of Hong Kong, Hong Kong, China; 2 Centre for Child and Family Science, The Education University of Hong Kong, Hong Kong, China; 3 Centre for Educational and Developmental Sciences, The Education University of Hong Kong, Hong Kong, China; 4 Department of Health Science, Ateneo de Manila University, Quezon City 1108, Philippines

**Keywords:** social support, parental correlates, sport, children and youth, review, physical activity, parental social influence

## Abstract

Parents are often regarded as one of the significant social agents who are important to the participation of physical activity (PA) among children and adolescents. However, within the literature, the relationships between parental influences and child and adolescent PA have been inconclusive and discordant. The purpose of this meta-analysis was to quantify and synthesize the associations between parental social influences (positive parental influence, punishment, and discouragement) and the PA level of children and adolescents. Through a systematic literature search using PsycINFO, Web of Science, PubMed, ProQuest, and SPORTDiscus databases, we identified 112 eligible studies and subsequently extracted 741 effect sizes for our analysis. Multilevel meta-analysis showed that the corrected zero-order correlation of positive parental influence was positive and statistically significant, r = 0.202, SE = 0.014, t = 14.975, *p* < 0.001, 95% confidence interval (CI) = [0.176, 0.228]. Further moderation analysis also found that this was significantly moderated by parental gender (maternal vs. paternal), respondent of influence measure (parent-reported vs. child-reported), and type of PA measure (subjective vs. objective). The corrected zero-order correlations of negative parental influences (i.e., punishment and discouragement) were not statistically significant, and no significant moderation effects were observed. The findings of our meta-analysis showed that children and adolescents had higher PA levels when their parents supported PA participation by exerting positive social influence. Punishment and discouragement against PA by parents did not appear to be significantly associated with the PA level of children and adolescents. The findings of negative parental social influence were mixed and required further investigations.

## 1. Introduction

Physical activity (PA) is well documented to benefit physical health [1,2,3], mental health [4], and social well-being [5]. However, physical inactivity among children and adolescents is now frequently reported worldwide [6]. As parents are often regarded as one of the significant social agents for promoting a physically active lifestyle among children and adolescents [7,8], as parent–child PA levels relate to social, environmental, psychological and demographic factors, including overweight/obesity [9], PA enjoyment, motivation, and self-sufficiency [10,11], school environment [12], neighborhood environments [13], etc. Our study aims to conduct a meta-analysis that synthesizes the research findings thus far regarding the relationship between parental influence and PA level of children and adolescents.

### 1.1. Positive and Negative Parental Influences

Parental influence on children’s PA encompasses multidimensional mechanisms, including parental attitudes, beliefs, values toward PA [14] and social support [15]. Parents are believed to exert their social influence on child and adolescent PA patterns through their encouragement [16], logistic support [8], role modeling [17], parent–child play [18], family communication [19] and general social support [20]. Parents may shape their children’s habits and actual involvement in PA by exerting influence in the sporting environment [21,22]. Given the importance and complexity of parental influence on PA, a large volume of studies have examined the role of parental support on child and adolescent PA [22,23,24]. It is generally found that the provision of positive parental support was associated with higher PA levels among children and adolescents.

Indeed, research regarding the relationship between parental influence and child PA has primarily focused on positive social influence from parents [22,23,24]. In comparison to positive parental influence, research into negative parental influence on child and adolescent PA has received far less attention in the literature. Negative parental influence is often exhibited in two forms: punishment and discouragement. Punishment is a negative parental influence characterized by forcing children to participate in PA or forcing them to perform better in sport/exercise by using coercive instruction styles, implementing excessive parental control, or applying pressure [25,26,27]. Discouragement is a negative type of parental influence that is defined as parental behaviors or verbalizations, such as disapproval of PA, restricting outside play or against participate in PA [28,29]. Within the limited pool of research, studies have generally reported mixed findings on the relationship between negative parental influences and PA levels of children and adolescents. Previous studies reported relationships that are either nonsignificant [25,29,30], positive [31], or negative [30,32], which could be dependent on the type of negative parental influences, the specification of the sample, and the measures of PA levels.

To better understand how parents can promote the PA levels of children and adolescents, it is important that research scrutinizes and synthesizes the discordant findings on negative parental influence, and compares the findings against those of positive parental influence.

### 1.2. Current Reviews about Parental Influence

Previous reviews [22,23,24] have summarized the research findings of how parental influences are related to child and adolescent PA levels. However, these studies mainly focus on positive parental influence, such as social support by parents. For example, the systematic review by Edwardson and Gorely [23] only covers studies related to social support from parents in terms of parental modeling, involvement, overall support, encouragement, and support in transportation. A systematic review by Beets and colleagues [22] investigates studies on social support from parents. Some of this was tangible support, such as supervision/accompaniment and instrumental support, and some was intangible support such as encouragement/praise, and provision of information. Yao and Rhodes [33] performed a meta-analysis on parental modeling and support. Despite differences in the conceptualization of social support, both reviews only looked at the positive side of parental influence on PA, and ignored aspects of negative parental influence such verbal pressure and restrictions. This precludes a complete understanding of parental influence on child and adolescent PA levels.

To the best of our knowledge, the only review in the literature that covers aspects of negative parental influence on PA levels is the systematic review by Lindsay and coworkers [34]. Their analysis [34] identified and discussed studies on how parents applied negative social influence on children’s and adolescents’ PA levels, such as implementation of rules and restrictions, and applying psychological control. The findings supported that there would be a negative or nonsignificant connection between negative parental influence and PA level. However, the review only included studies on Latino children in the United States. Moreover, their conceptualization of negative parental influence was restricted to how parents hindered or prohibited children from taking part in PA, and did not include promoting PA in a coercive way [34].

In conclusion, existing reviews reveal a wealth of literature investigating parental influence on children’s and adolescents’ PA. However, these reviews do not examine whether parents promote PA to their children using positive or negative techniques. Moreover, the findings from these systematic reviews are unable to statistically quantify the effect sizes of the relationship between parental influence and PA. Furthermore, potential moderators of such effects, such as age, gender, parental gender, and type of PA measures, remain unresearched.

### 1.3. The Present Study

To address the research gaps in the literature, we present a meta-analysis that synthesizes the findings on the extent to which positive and negative parental influence are related to the PA level of children and adolescents. Based on the key findings of the literature on positive and negative parental influences [27,35] and the evidence from systematic reviews [22,23,24], we would examine the following research questions:

(Q1) What is the association between positive parental influence and PA level of children and adolescents?

(Q2) What is the association between negative parental influence styles of punishment and discouragement, and PA level of children and adolescents?

(Q3) Whether the associations identified in Q1 and Q2 would hold in different study characteristics, such as child age group, child gender, parental gender, respondent of parental influence, and the type of PA measurement.

## 2. Materials and Methods

### 2.1. Literature Search

We used the Preferred Reporting Items for Systematic Reviews and Meta-Analyses (PRISMA) checklist [36] as a guide to conduct this meta-analysis. Descriptions, aims and hypotheses of this meta-analysis were preregistered at PROSPERO with ID CRD42021267072. This review article focuses on the relationships between parental influence and the PA levels of children and adolescents. A literature search was conducted in April 2020 using PsycINFO, Web of Science, PubMed, ProQuest, and SPORTDiscus databases. A combination of Boolean keywords related to the PA and parental influence of healthy children and adolescents were used as the search terms. The specific keywords can be found in File S1 in the online supplementary material.

### 2.2. Inclusion and Exclusion Criteria

Our systematic search aimed to identify all studies published up to April 2020 that met the inclusion and exclusion criteria. Studies were included if:

1. sample measured children or adolescents under 18 years of age;
2. sample were healthy individuals (i.e., no known physical or mental conditions);
3. PA level was measured;
4. positive or negative PA-specific parental social influence was measured;
5. they were quantitative in nature;
6. they were published in English peer-reviewed journals;

We excluded opinion articles, reviews, commentaries, and unpublished papers (e.g., student theses) from our review.

### 2.3. Search and Data Extraction Procedure

**Figure 1:**
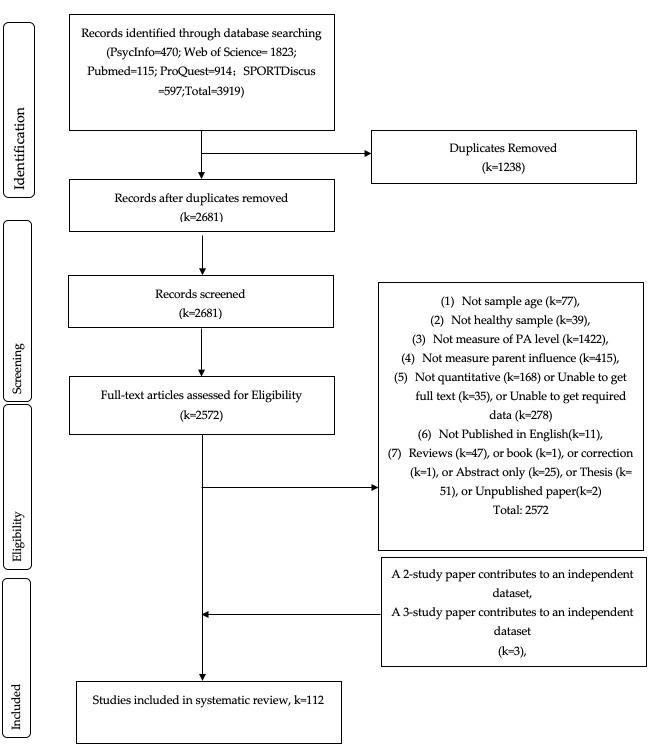
Preferred Reporting Items for Systematic Reviews and Meta-Analyses (PRISMA) flowchart used to identify studies for detailed analysis of parental influence and PA level.

In the initial search, 3919 articles were identified. After removing 1238 duplications, a total of 2681 records remained for title-and-abstract screening based on the inclusion and exclusion criteria. After excluding 2572 studies (reasons are displayed in the PRISMA diagram of Figure 1), a total of 112 studies remained for the current meta-analysis with 714 effect sizes (i.e., zero-order correlation coefficients) extracted regarding the relationships between parental influence and PA levels of children and adolescents.

### 2.4. Classification and Study Quality Assessment

Apart from the effect sizes, two independent coders extracted key study variables and assessed study quality. The study characteristics were coded according to:

- parental influence type (i.e., positive influence vs. punishment vs. discouragement)
- child age group (i.e., children vs. adolescents)
- child gender (i.e., male vs. female)
- parental gender (maternal vs. paternal)
- respondent of parental influence (i.e., child-reported vs. parent-reported)
- type of PA measurement (i.e., objective vs. subjective)

Specific definitions of the classifications can be found in File S2 (online supplementary material). For the assessment of study quality, we applied the Revised Risk of Bias Assessment [37] tool by Ntoumanis and colleagues [38]. The study was considered to be either ‘low risk’ or ‘having a potential risk of bias’, depending on whether the study could fulfil the 15 assessment criteria of study quality outlined by the tool [38]. Two raters discussed and resolved any disagreement in their classifications and scoring until a consensus was reached.

### 2.5. Multilevel Meta-Analysis

We conducted a multilevel meta-analysis using the Metafor package [39] in R and RStudio [40]. This method was suitable for our study because we had to extract multiple effect sizes from a single study that had more than one measure of parental influence or PA level. We were also able to statistically control for the nested effects of sample dependency between related effect sizes. In this case, our analysis did not violate the assumption of independent observations from traditional univariate meta-analysis, and more importantly, we were able to achieve higher statistical power by maximizing the available information.

In particular, our multilevel meta-analysis followed a three-level random-effects model [41]:

- at level 1, we accounted for sampling variance (participant sampling);
- at level 2, we accounted for within-study variance;
- at level 3, we accounted for between-study variance [42,43].

We examined Q1 and Q2 in 2 steps. In Step 1, we examined the overall effect sizes. In Step 2, we tested heterogeneity of overall effect sizes by applying a likelihood-ratio test based on the distribution of within-study variance, between-study significance, and sampling variance over the three levels of our meta-analytic model. If less than 75% of the total variance could be attributed to the sampling variance, we proceeded to Step 3, where we tested H3. Here, we conducted moderation analysis to examine whether the overall effect sizes were moderated by our coded classification of the study characteristics/effect sizes [44]. As Fisher’s z is the default effect size for the multilevel meta-analysis using the Metafor package [39], we followed the procedures of previous studies [45,46] in converting zero-order correlations to Fisher’s z for the analysis, and vice versa to simplify the interpretation of study findings.

In addition to multilevel meta-analysis, we also evaluated whether publication bias [47,48] inflated our meta-analyzed effect sizes by conducting Egger’s test and funnel plot using Fisher’s z transformations [49]. A significant Egger’s test statistic and an asymmetrical funnel plot will indicate a presented risk of publication bias, and therefore, more cautious interpretation would be needed.

## 3. Results

### 3.1. Quality of Studies

The majority of included studies (k = 72) were rated low risk in each assessment item and 40 studies had potential risks of biases (18 studies concerning the method, 22 studies concerning the results section). More detailed item-by-item ratings can be found in File S3.

### 3.2. Descriptive Statistics

A total of 112 studies and 714 effect sizes were included in the current meta-analysis. The total sample size was *n* = 943,448, with study sample sizes ranging from *n* = 30 [50] to *n* = 81,857 [51]. The overall sample mean age was 10.91 years old. The majority of studies (k = 95) were cross-sectional in design, and other studies were longitudinal research (k = 15), experimental or intervention studies (k = 1) and prospective studies (k = 1). The majority of the literature focused on positive influence (k = 111), and only a few studies (k = 5) were related to the punishment of parental influence and to the discouragement of parental influence (k = 4). There were studies that included both males and females as children and adolescent samples (k = 91), while some studies (k = 12) only looked specifically at female samples. There was no study focusing on a male-only sample and only a few studies did not report gender proportion (k = 9). In terms of parental gender, most studies adopted the concept of parents without distinguishing between paternal and maternal influence (k = 76), while some studies concentrated on paternal influence (k = 31), and others focused on maternal influence (k = 35). For the coding of respondent of influence measure, there were slightly more studies (k = 71) using parent-reported than used child-reported parental influence (k = 50). Studies were also coded with subjective measurements (k = 84) and objective measurements (k = 35) of PA.

### 3.3. Publication Bias

**Figure 2:**
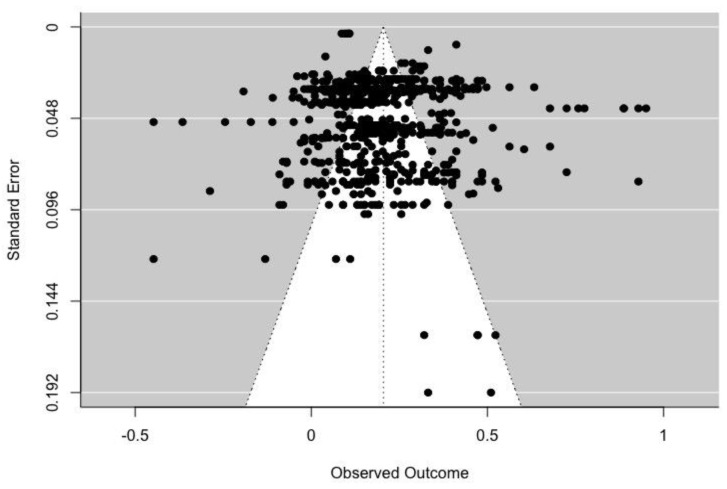
Funnel plot for the associations between positive parental influence and PA level.

**Figure 3:**
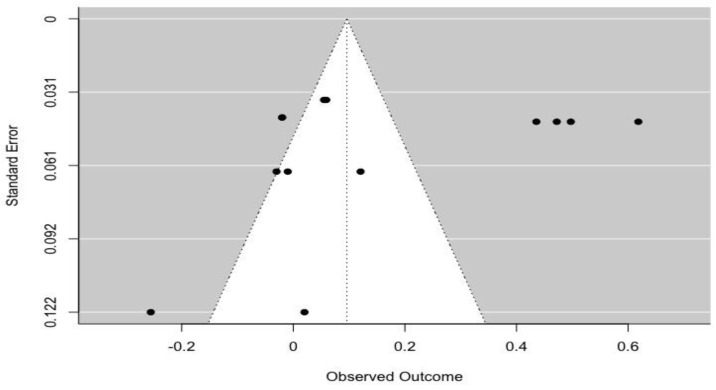
Funnel plot for the associations between punishment parental influence and PA level.

**Figure 4:**
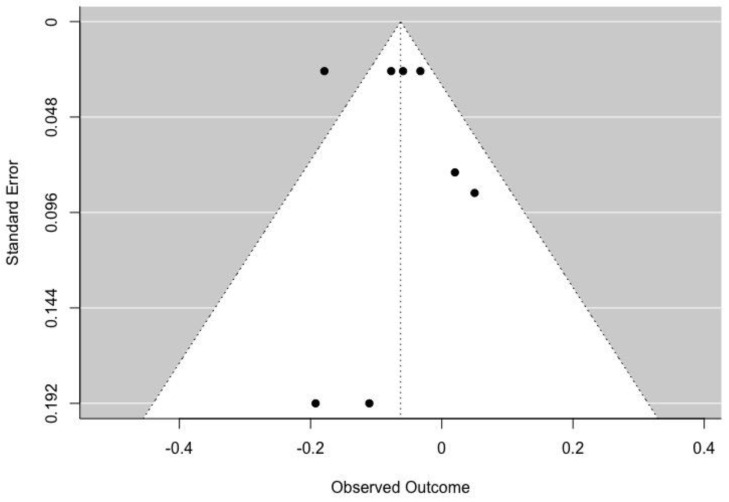
Funnel plot for the associations between discouragement parental influence and PA level.

Egger’s tests indicated that no significant publication bias was detected in positive parental influence (z = 0.144, *p* = 0.886), punishment (z = −1.757, *p* = 0.079), and discouragement (z = 0.372, *p* = 0.710). Figure 2, Figure 3 and Figure 4 illustrate the associations between PA level and positive, punishment and discouragement parental influence, respectively.

### 3.4. Main Overall Effect (Q1 and Q2)

#### 3.4.1. Overall Effect Size of Positive Parental Influence

The main overall effect size of positive influence was statistically significant (r = 0.202, SE = 0.014, t = 14.975, *p* < 0.001, 95% CI = [0.176, 0.228]), with substantial heterogeneity (Q_E_(686) = 12,259.262, *p* < 0.001). The variance at the within-study level (*p* < 0.001) and the between-study level (*p* < 0.001) were both significant. Follow-up analyses concluded that variance at the sampling, within-study, and between-study levels was 3.02%, 33.00%, and 63.97%, respectively. Since the percentage of total variance attributed at level 1 is less than 75%, further moderation analysis is meaningful [44].

#### 3.4.2. Overall Effect Size of Punishment

The main overall effect size of the punishment did not reach statistical significance (r = 0.096, SE = 0.109, t = 0.881, *p* = 0.396, 95% CI = [−0.141, 0.322]), with substantial heterogeneity (Q_E_(12) = 348.475, *p* < 0.001). The variance did not reach significance *p* = 0.182 at the within-study level; however, it reached significance (*p* < 0.001) at the between-study level. Follow-up analyses concluded that variance at the sampling level, within-study level and between-study level was 1.32%, 3.39%, and 95.28%, respectively. Because the proportion of total variation ascribed at the sampling level is less than 75%, further conduction of moderation analysis is meaningful [44].

#### 3.4.3. Overall Effect Size of in Discouragement

The overall effect size regarding discouragement failed to reach statistical significance (r = −0.063, SE = 0.035, t = −1.789, *p* = 0.117, 95% CI = [−0.145, 0.020]), with substantial heterogeneity (Q_E_(7) = 24.381, *p* < 0.001). The variance at the within-study level *p* < 0.001 and the between-study level *p* < 0.001 were both significant. Follow-up analyses concluded that variance at the sampling level, within-study level and between-study level was 1.29%, 68.42%, and 15.40%, respectively. Potential meaningful moderation analysis could be conducted because the percentage of total variance attributed at the sampling level is less than 75% [44].

### 3.5. Moderator Analysis (Q3)

#### 3.5.1. Sample Demographics

First, we tested demographic moderators of age and gender in positive, punishment and discouragement influence models. Neither age (Q_E_(489) = 8082.320, *p* < 0.001, F(1, 489) = 0.253, *p* = 0.615) nor gender (Q_E_(653) = 12,088.451, *p* < 0.001, F(1, 653) = 0.604, *p* = 0.437) was a significant moderator of the relationship between positive parental influence and PA level. The same was observed in punishment, where age (Q_E_(11) = 228.024, *p* < 0.001, F(1, 11) = 1.297, *p* = 0.279) and gender (Q_E_(11) = 346.680, *p* < 0.001, F(1, 11) = 0.412, *p* = 0.534) did not significantly moderate the relationship. Similar patterns were also found in discouragement, where age (Q_E_(5) = 22.498, *p* < 0.001, F(1, 5) = 0.077, *p* = 0.792) and gender (Q_E_(5) = 22.532, *p* < 0.001, F(1, 5) = 0.144, *p* = 0.720) did not significantly moderate the relationship between discouragement and PA level. This suggests that the relationship between parental influence (positive influence/punishment/discouragement) and PA remained stable across different ages and genders of children.

#### 3.5.2. Parental Gender

Parental gender had two categories. Effect sizes of maternal/paternal influence were accounted for in the positive influence (k = 30)/(k = 34), punishment (k = 1)/(k = 1) and discouragement (k = 0)/(k = 0). For the relationship between positive parental influence and PA level, parental gender was a significant moderator (Q_E_(251) = 1773.112, *p* < 0.001, F(2, 251) = 55.907, *p* < 0.001,). However, paternal influence (β = 0.203, S.E. = 0.020, t = 10.565, *p* < 0.001, 95% CI = [0.168, 0.240]) showed similar effects to maternal influence (β = 0.180, S.E. = 0.019, t = 9.460, *p* < 0.001, 95% CI = [0.143, 0.217]), indicating significance. For the relationship between punishment and PA level, parental gender did not moderate the effect between parental influence and PA level (Q_E_(0) = 0.000, *p* = 1.000, F(2, 1) = 2.199, *p* = 0.43). For the relationship between discouragement and PA level, there were not enough studies that distinguished between paternal-only and maternal-only influence, so we were unable to conduct a moderation analysis for discouragement.

#### 3.5.3. Respondent of Parental Influence

For respondent of parental influence, the effect sizes of parent-reported measures/child-reported measures were accounted for in the positive influence (k = 53)/(k = 47), punishment (k = 10)/(k = 3) and discouragement (k = 8)/(k = 0), respectively, for moderation analysis. For the relationship between positive parental influence and PA level, respondent of parental influence was a significant moderator (Q_E_(685) = 11,227.767, *p* < 0.001, F(2, 685) = 116.372, *p* < 0.001). Both child-reported measures and parent-reported measures were significant. However, compared with child-reported measures (β = 0.148, S.E. = 0.017, t = 8.641, *p* < 0.001, 95% CI = [0.114, 0.181]), parent-reported measures had significantly stronger positive correlations with PA level (β = 0.235, S.E. = 0.016, t = 15.239, *p* < 0.001, 95% CI = [0.206, 0.265]). For the relationship between punishment and PA level, the respondent of parental influence was not found to be a significant moderator (Q_E_(11) = 323.908, *p* < 0.001, F(2, 11) = 0.330, *p* = 0.726). For the relationship between discouragement and PA level, we were unable to conduct a moderation analysis due to an inadequate number of relevant effect sizes.

#### 3.5.4. Type of PA Measure

For type of PA measure moderator, effect sizes of subjective measurement methods/objective measurement methods were accounted for in the positive influence (k = 21)/(k = 77), punishment (k = 11)/(k = 2) and discouragement(k = 3)/(k = 5), respectively. For the relationship between positive parental influence and PA, the type of PA measure was a significant moderator (Q_E_(685) = 12,257.973, *p* < 0.001, F(2, 685) = 121.879, *p* < 0.001). Both subjective (β = 0.218, S.E. = 0.015, t = 15.135, *p* < 0.001, 95% CI = [0.191, 0.246]) and objective measures (β = 0.154, S.E. = 0.022, t = 7.118, *p* < 0.001, 95% CI = [0.113, 0.195]) were significant moderators. On the other hand, the type of PA measurement did not significantly moderate the relationship between punishment and PA (Q_E_(11) = 334.874, *p* < 0.001, F(2, 11) = 0.786, *p* = 0.480) and the relationship between discouragement and PA (Q_E_(6) = 23.499, *p* < 0.001, F(2, 6) = 0.645, *p* = 0.557).

## 4. Discussion

This three-level meta-analysis aimed to synthesize the findings of the literature regarding the extent to which positive and negative parental influences, such as positive influence, punishment and discouragement, were related to the PA levels of children and adolescents (under the age of 18). A total of 112 studies and 714 effect sizes were analyzed based on the total sample size of *n* = 943,448.

Our meta-analyzed associations may answer the three research questions regarding the relationships between parental influence and children’s PA level. For Q1, positive parental influence was positively and significantly associated with the PA level of children and adolescents. For Q2, the two negative parental influences of punishment and discouragement did not significantly link to the PA level of children and adolescents. For Q3, the association between positive parental influence and PA level was significantly moderated by parental gender, respondent of parental influence measure, and type of PA measure.

In sum, the findings showed that the relationship between parental influence and the PA level of children and adolescents was dependent on the type of parental influence that children are subject to. The role of positive parental influence was shown to be adaptive and robust, but that of negative parental influence, i.e., punishment and discouragement- appeared to be nonsignificant.

### 4.1. Positive Parental Influence

Our results show that positive parental influences are positively related to child and adolescent PA levels with small to medium effects. This is consistent with the findings of previous meta-analyses that investigated the support of parents on children’s and adolescents’ PA levels [33,52]. Moderation analyses of our meta-analyzed effect sizes also showed that the correlation between positive parental support and PA level was generally consistent in different sampling characteristics such as child age group and child gender. This pattern of results concurs with the findings of previous systematic reviews [23,24] and a previous study about PA and child gender [53]. This may indicate that positive parental influence is equally important for children and adolescents of both genders with regard to PA participation. However, a few significant moderation effects were observed in other study characteristics: parental gender, respondent of parental influence, and the type of PA measurement. These warrant further discussion.

### 4.2. Moderators of Positive Parental Influence

Our findings show that parental gender significantly moderates the relationship between positive parental influence and PA level, but is not different between mothers and fathers. This is in agreement with the findings of the meta-analysis by Laird and colleagues [52]. As such, our findings do not concur with the view that fathers and mothers have different roles in influencing their children’s participation in PA [54] or sports [35]. This could be the case since both parents are accountable for supporting their children’s physical activity [55].

Moderation analysis shows that positive parental influence is more significantly correlated with PA level when the evaluation is parent-reported than when it is child-reported. It is plausible that parents have a better understanding of the social support they offer to their children in terms of PA participation [56], resulting in a stronger correlation with PA level due to reduced measurement error.

Nevertheless, our moderation analysis shows that there is no significant difference when measuring PA objectively versus subjectively in the relationship between positive parental influence and PA, although this finding contradicts the theory that participants may overestimate or underestimate the amount of PA [57]. One possibility is that individuals are able to properly recall their level of PA, especially with age increase [58], and evidence shows that the self-report and accelerometer data are moderate to highly correlated [59].

Overall, our moderation analyses have shown that the positive relationship between positive parental influence and the PA level of children and adolescents is generally robust against the variation in sample/study characteristics. However, the respondent of parental influence appears to affect the strength of the relationship.

### 4.3. Negative Parental Influences and Moderation

In terms of negative parental influence, our meta-analysis showed that punishment and discouragement did not significantly correlate with the PA level of children and adolescents. These findings conflict with the literature’s general perspective that controlling parenting styles or negative parenting practices, such as rule setting and psychological control, can discourage children’s motivational and behavioral patterns of PA [34,60,61].

Similarly, it has been argued in the literature that negative parental influence, such as punishment or negative feedback, can be perceived as constructive criticism [35,62]. Adolescents are more likely to perceive this than children as they are more cognitively mature and thus can understand the motivation behind the criticism [35]. Indeed, our study does not reveal a significant moderation effect of age on the relationship between punishment and PA level; however, this could be because of the small sample size of punishment-related studies (k = 5), which reduced the statistical power of the moderation analysis. Similarly, the nonsignificant relationship between discouragement and PA level we found could also be attributed to the small sample size of discouragement-related studies (k = 4). Such a finding could imply that children’s PA level is unlikely to be reduced by parental disapproval. Perhaps it was that our meta-analysis did not differentiate where PA was taken place, so the role of parental discouragement on PA could be different between school-based or leisure-time PA. It is therefore important that studies take the influence of school or PE teachers into account when they evaluate the role of parents on child PA levels [27,35].

Overall, it appears that the research findings regarding the role of negative parental influence on children’s PA levels are mixed and inconclusive. As such, future studies should investigate how parental punishment and discouragement impact the volume and behavioural patterns of children’s PA both in school and out of school.

### 4.4. Limitations and Future Directions

First, our investigation only focuses on the association between parental influence and PA level of children and adolescents. The majority of the studies we identified from the literature were cross-sectional. Without longitudinal studies, the meta-analyzed correlations reported in our study are unable to make any causal or temporal inferences.

Second, the sample sizes of certain subgroups that we coded for our moderation analyses were relatively small. This is because the included studies were often not able to differentiate the effect sizes between the categories (e.g., paternal vs. maternal influence), or the number of studies that fell within the coding classifications of certain moderators was limited (e.g., discouragement). As such, small sample sizes reduced the statistical power of our meta-analysis to detect significant differences between subgroups. They also precluded our ability to conduct moderation analysis for interactive moderation effects of two or more moderators.

Third, a large volume of studies examines the parental influence on children’s psychological patterns of PA participation, e.g., enjoyment [63], motivation [64], intention [65], and commitment [66]. However, these were excluded from our meta-analysis because they did not have a behavioral measure of PA level. Therefore, our findings were exclusive to how parental influence was linked to PA level, instead of the psychological patterns of children and adolescents in PA contexts. Future meta-analyses or systematic reviews should synthesize the research findings of parental influence and psychological factors of PA participation in children and adolescents.

Last, the mixed findings of negative parental influence may suggest that more studies are required to scrutinise the role of parental negative influences on children and adolescents from PA. The literature has documented that parental concerns about safety, availability of time, and the importance of academic performance as common barriers that prevent children and adolescents from participating in PA [67,68,69]. It is highly important that future studies examine how parents cope with these barriers and preserve the PA level of their children.

## 5. Conclusions

This meta-analysis is a comprehensive summary of the association between parental influence and the PA level of children and adolescents. To the best of our knowledge, this is the first meta-analysis in the literature that focuses on both positive and negative parental influences, and their associations with the PA level of children and adolescents. Our findings support the view that the PA level of children and adolescents is more likely to be higher when they receive approval, support/assistance, and recognition/reward from their parents, which answered Q1. In Q2, there was no significant correlation between children’s and adolescents’ PA levels and the two negative parental influence of punishment and discouragement. Parental gender, the responder of the parental influence measure, and the kind of PA measure all significantly moderated the link between positive parental influence and PA level for Q3. Our current study may serve as a foundation to understand how parents and their influence types may optimise or impair PA behaviors.

## Data Availability

Not applicable.

## Supplementary Materials

The following supporting information can be downloaded at: https://www.mdpi.com/article/10.3390/ijerph192416861/s1, File S1: Search String; File S2: Table S1: Positive parental influence definitions and example of questionnaire items; Table S2: Punishment parental influence definitions and example of questionnaire items; Table S3: Discouragement parental influence definitions and example of questionnaire items; File S3: Study Quality, Table S4. Risk of Bias Assessment Criterion Items; Table S5. Risk of Bias Assessment Results. References [70,71,72,73,74,75,76,77,78,79,80,81,82,83,84,85,86] are cited in the supplementary materials.
