# Supplementary material for: Parental Influence on Child and Adolescent Physical Activity Level: A Meta-Analysis
Source: Int J Environ Res Public Health. 2022 Dec 15;19(24):16861. doi: 10.3390/ijerph192416861 (PMC9778652; doi:10.3390/ijerph192416861)
Supplement: Supplementary file 1 [file ijerph-19-16861-s001.zip › Appendix/Appendix A.pdf]

International Journal of
Environmental Research and Public Health

MDPI

Appendix A

Search String

The below search string was searched in PsycInfo, Web of Science, Pubmed, ProQuest and SPORTDiscus without a year limit, and only filters of English were used.

"physical* activit*" OR "exercise" OR "sport" OR "movement*" OR "fitness" OR "moderate to vigorous PA"【AND】"father" OR "mother" OR "parent*" OR "paternal" OR "maternal"【OR】"reinforcement" OR "affiliation" OR "punishment" OR "dysfunction" OR "parent* encouragement" OR "social support" OR "social influence" OR "role of significant others" OR "determinants"【OR】"parental support" OR "parental role" OR "parent* influence*" OR "perceived support from parent" OR "parent's reports of direct support" OR "parental promot*" OR "parental correlates"【OR】"support from family" OR "family support" OR "family social support" OR "activity-related support" OR "parental restriction" OR "parental perceptions" 【AND】"child*" OR "adolescen*" OR "youth" OR "student*" OR "young" OR "school-age" OR "teenage*" OR "preadolescent" OR "preschool*" OR "schoolchildren"【NOT】"disorder" OR "autism" OR "cancer" OR "diabetes" OR "disabilit*"
.
