# Supplementary material for: Parental Influence on Child and Adolescent Physical Activity Level: A Meta-Analysis
Source: Int J Environ Res Public Health. 2022 Dec 15;19(24):16861. doi: 10.3390/ijerph192416861 (PMC9778652; doi:10.3390/ijerph192416861)
Supplement: Supplementary file 1 [file ijerph-19-16861-s001.zip › Appendix/Appendix B .pdf]

International Journal of
Environmental Research and Public Health

MDPI

Appendix B

Table A1 Positive parental influence definitions and example of questionnaire items

Positive parental influence	Definitions	Example
**Encouragement**	Parents' language or behavior to enhance the motivation of children's physical activity participation or to keep fit [1].	"How much does your mother/guardian encourage you to be physically active?"[1]
**Role modeling**	Parents' high level of PA serving as an example for children and adolescents to be physically active [2].	"How often does your child see you being physically active?"[2]
**General social support**	Containing multiple types of support, including tangible support [3, 4], intangible support [3, 4], logistic support [2], etc.	"How often do you discuss benefits being physically active?"[4]
**Appropriateness**	A view or belief perceived by children and adolescents, to which extent significant others think PA support was appropriate [5].	"How do you think the following people feel about the appropriateness of competitive sport participation for adolescent females in general? "[5]
**Help**	The frequency of perceived direct parental help towards organizing exercises [6].	"Direct help in exercising hard was assessed by asking about frequency of help from parents in organizing exercise sessions of physical activities." [6]
**Value**	A belief hold by children and adolescents about how valuable or important their parents thought to be physically active or fit [6].	The value of physical activity of significant others was covered through questions regarding the importance parents and friends place on being physically fit and the importance friends place on being a good athlete." [6]
**Play with**	It is about co-participation and participating in physical activities together. Parents might choose to participate in sports with their children or share PA with them in	"How many days in the past week did you do a physical activity with either of your parents?"[8]

	order to raise their children's PA levels [7].	
**Transportation**	Picking up children when they need to go to physical education classes or sports activities or parents facilitated their children with transportation to participate in sports, especially those held outside school [9].	"How much does your mother/father provided transportation to be active for you? "[10]
**Emotional support**	It is referring to emotional aid, encouragement, stimulation of participation and praise [11-13].	"How often does your parent or guardian encourage you to participate in regular physical activity?"[12]
**Monitoring/watch**	Supervising child's exercise to ensure the quality, amount or safety of training [14].	"How much do you keep track of the amount of physical activity/active play/sport your child is getting?"[14, 15]
**Reinforcement**	Also known as praise, it is referred to recognition of child's performances and efforts [14].	"How often do you praise your child for being physically active? "[14]
**Allow**	Giving permission for children and adolescents to play outside in the neighborhood [16].	"How often do you allow child to play out anywhere in neighborhood? "[16]
**Autonomy support**	It is related to an atmosphere, having freedom or rights to talk or choose of being physical active based on the willingness of children [17].	"I feel that my parents provide me with choices, options, and opportunities about whether to do active sports and/ or vigorous exercise in my free time."[18, 19]
**Talk / Knowledge-sharing**	It is about kids being able to express or understand issues or feelings during PA session [20], and this support was also related to parents talking or comments towards the performance or sharing knowledge/ beneficial effects of PA with child [17].	"How often do your parents tell you that you are doing well in physical activity or sports?"[21]
**Fees/Financial support**	Paying fees of lessons related to physical activity or organized physical activity [17].	"Do you provide financial support when your child participates in physical activity/exercise? "[17]

**Facilitation**	Is also defined as logistic support [2].	"Do you facilitate your child in participating in sports or other activities that stimulate PA?"[2]
**Accompanying**	Staying with children when they exercise [17].	"Do you accompany your child when he/she attends physical activity/exercise sessions?"[17]
**Plan**	Detailed arrangement (e.g., time, place, frequency, contents) in advance to ensure children have adequate amount of movement or exercise [22].	"How often to engage my child in at least 3 h of physical activity every day over the next week."[22]
**Stimulation**	Guiding and leading children's PA behaviors to be active [23].	"I make sure that my child travels actively on foot or by bicycle (with or without me) as often as possible."[23]
**Collaborative social control**	A tactic parents would employ to increase children PA level by being role models for children [23].	"How likely is it that you would participate in physical activity so the child could see it as a means of increasing your child's physical activity?"[23]
**Positive social control**	A control strategy (e.g., encouragement, or talking about pleasure of being active) adopted by parents [23].	"How likely is it that you would encourage your child to stick with his/her physical activity?"[23]

Table A2 Punishment parental influence definitions and example of questionnaire items

Punishment parental influence	Definitions	Example of question items
**Pressure**	Verbally stimulate children to exercise more [24].	"Suggest that I lose weight."[24]
**Control / Negative social control**	Nag or strong rules captured by parents to achieve goals of letting their kids being physically active [23].	"How likely is it that you would nag your child to be active as a means of increasing your child's physical activity?"[23]

Table A3 Discouragement parental influence definitions and example of questionnaire items

Discouragement parental influence	Definitions	Example of question items

**Discouragement**	The opposite way of encouragement. Parents may suppress children PA attempt by certain behaviors and language [25]	An observation system capture "restrains from action, redirects or moves in another direction, pushes, spanks."[25]
**Restrictions / Constraints / Rules**	Prevent or limit children's outdoor activity and potential risky behaviors (e.g., playing rough games, climbing trees) due to safety concern [15, 26]	"Do not play outside without permission or Stay close to or within sight of the house/parent."[15, 26]

References

1. J. M. DiNallo, J. F. Savage and D. S. Downs. Adolescent body satisfaction: The role of perceived parental encouragement for physical activity. *J. Sport Exerc. Psy.* **2007**, *29*, S158-S159.
2. D. H. H. Van Kann, S. P. J. Kremers, S. I. de Vries, N. K. de Vries and M. W. J. Jansen. Parental active transportation routines (PATRns) as a moderator of the association between neighborhood characteristics and parental influences and active school transportation. *Environ. Behav.* **2016**, *48*, 946-965.
3. E. K. Wing, M. Bélanger and J. Brunet. Linking parental influences and youth participation in physical activity in- and out-of-school: The mediating role of self-efficacy and enjoyment. *Am. J. Health Behav.* **2016**, *40*, 31-37.
4. H. Qurban, J. Wang, H. Siddique, T. Morris and Z. Qiao. The mediating role of parental support: The relation between sports participation, self-esteem, and motivation for sports among chinese students. *Curr. Psychol.* **2019**, *38*, 308-319.
5. B. A. Brown, B. G. Frankel and M. P. Fennell. Hugs or shrugs: Parental and peer influence on continuity of involvement in sport by female adolescents. *Sex Roles* **1989**, *20*, 397-412.
6. N. Anderssen and B. Wold. Parental and peer influences on leisure-time physical activity in young adolescents. *Res. Q. Exerc. Sport* **1992**, *63*, 341-348.
7. R. R. Rosenkranz and D. A. Dzewaltowski. Maternal physical-activity-related parenting behaviors may influence children's physical activity levels and relative weight. *Women Sport Phys Act J* **2011**, *20*, 3-12.
8. S. M. Price, J. McDivitt, D. Weber, L. S. Wolff, H. A. Massett and J. E. Fulton. Correlates of weight-bearing physical activity among adolescent girls: Results from a national survey of girls and their parents. *J Phys Act Health* **2008**, *5*, 132-145.
9. W. R. Hoefer, T. L. McKenzie, J. F. Sallis, S. J. Marshall and T. L. Conway. Parental provision of transportation for adolescent physical activity. *Am. J. Prev. Med.* **2001**, *21*, 48-51.
10. P. Wilk, A. F. Clark, A. Maltby, P. Tucker and J. A. Gilliland. Exploring the effect of parental influence on children's physical activity: The mediating role of children's perceptions of parental support. *Prev. Med.* **2018**, *106*, 79-85.
11. R. Vilhjalmsson and T. Thorlindsson. Factors related to physical activity: A study of adolescents. *Soc. Sci. Med* **1998**, *47*, 665-675.
12. C. M. Sabiston and P. R. E. Crocker. Exploring self-perceptions and social influences as correlates of adolescent leisure-time physical activity. *J. Sport Exerc. Psy.* **2008**, *30*, 3-22.
13. L. E. Huffman, D. K. Wilson, M. L. Van Horn and R. R. Pate. Associations between parenting factors, motivation, and physical activity in overweight African American adolescents. *Ann. Behav. Med.* **2018**, *52*, 93-105.

14. E. M. Arredondo, J. P. Elder, G. X. Ayala, N. Campbell, B. Baquero and S. Duerksen. Is parenting style related to children's healthy eating and physical activity in Latino families? *Health Educ. Res.* **2006**, *21*, 862-871.

15. P. D. Loprinzi, D. P. Schary, M. W. Beets, J. Leary and B. J. Cardinal. Association between hypothesized parental influences and preschool children's physical activity behavior. *Am. J. Health Educ.* **2013**, *44*, 9-18.

16. A. M. McMinn, S. J. Griffin, A. P. Jones and E. M. F. van Sluijs. Family and home influences on children's after-school and weekend physical activity. *Eur. J. Public Health* **2013**, *23*, 805-810.

17. Y. Liu, Y. Zhang, S. Chen, J. Zhang, Z. Guo and P. Chen. Associations between parental support for physical activity and moderate-to-vigorous physical activity among Chinese school children: A cross-sectional study. *J. Sport Health Sci.* **2017**, *6*, 410-415.

18. L. McDavid, A. E. Cox and A. J. Amorose. The relative roles of physical education teachers and parents in adolescents' leisure-time physical activity motivation and behavior. *Psychol. Sport Exerc.* **2012**, *13*, 99-107.

19. D. Gonzalez-Cutre, A. Sicilia, M. Beas-Jimenez and M. S. Hagger. Broadening the trans-contextual model of motivation: A study with Spanish adolescents. *Scand. J. Med. Sci. Sports* **2014**, *24*, E306-E319.

20. M. A. Kobayashi, T. K. Lee, R. O. Leite, B. N. Esquives, G. Prado, S. E. Messiah and S. M. St George. The effects of parental stress on physical activity among overweight and obese hispanic adolescents: Moderating role of family communication and gender. *J Phys Act Health* **2019**, *16*, 952-961.

21. D. Sanz-Martín. Relationship between physical activity in children and perceived support: A case studies. *Apunt. Educ. Fis. y Deportes.* **2020**, 19-26.

22. K. Hamilton and R. Schwarzer. Parents' planning for physical activity for their pre-school aged children: The role of psycho-social mediators and moderators. *J. Child Fam. Stud.* **2018**, *27*, 421-430.

23. N. Liszewska, U. Scholz, T. Radtke, K. Horodyska, M. Liszewski and A. Luszczynska. Association between children's physical activity and parental practices enhancing children's physical activity: The moderating effects of children's BMI z-score. *Frontiers in Psychology* **2018**, *8*,

24. K. Zarychta, B. Mullan and A. Luszczynska. It doesn't matter what they say, it matters how they behave: Parental influences and changes in body mass among overweight and obese adolescents. *Appetite* **2016**, *96*, 47-55.

25. R. C. Klesges, J. M. Malott, P. F. Boschee and J. M. Weber. The effects of parental influences on children's food intake, physical activity, and relative weight. *Int. J. Eat. Disord.* **1986**, *5*, 335-346.

26. N. C. Crespo, K. Corder, S. Marshall, G. J. Norman, K. Patrick, J. F. Sallis and J. P. Elder. An examination of multilevel factors that may explain gender differences in children's physical activity. *J Phys Act Health* **2013**, *10*, 982-992.
