# Supplementary material for: Parental Influence on Child and Adolescent Physical Activity Level: A Meta-Analysis
Source: Int J Environ Res Public Health. 2022 Dec 15;19(24):16861. doi: 10.3390/ijerph192416861 (PMC9778652; doi:10.3390/ijerph192416861)
Supplement: Supplementary file 1 [file ijerph-19-16861-s001.zip › Appendix/Appendix C.pdf]

International Journal of
Environmental Research and Public Health

MDPI

Appendix C

Study Quality

Table A4 Risk of Bias Assessment Criterion Items

	**Risk of Bias Assessment Criterion**
1	Participants are randomly selected
2	Sample sizes are adequate
3	Participants are representative of various demographic groups
4	Exclusion of participants and justification
5	Group comparisons are made on other meaningful demographics
6	Validated measures are used or authors have provided sufficient supportive informaiton on psychometric properties they devised
7	Measures used were defined and appropriate
8	Authors have examined whether dropouts are random or not (longitudinal/prospective study)
9	Drop-out rate was not high and missing data was treated appropriately (longitudinal/prospective design)
10	allocation sequence generated to produce comparable groups (experimental design only)
11	allocation was concealed (experimental design only)
12	whether blinding was done; and if so, whether it was effective (experimental design only)
13	Outcome data for all outcomes were reported; incomplete outcomes due to attrition and exclusions were addressed (experimental design)
14	no selective outcome reporting (experimental design)
15	Other sources of bias (experimental design)

Table A5 Risk of Bias Assessment Results

Risk of Bias Assessment															
Author（year）	1	2	3	4	5	6	7	8	9	10	11	12	13	14	15	Overall assessment result
Klesges et al. (1986)	yes	yes	yes	yes	yes	yes	yes	na	na	na	na	na	na	na	na	low risk
Sallis et al. (1988)	yes	yes	yes	yes	yes	yes	yes	yes	yes	yes	yes	yes	yes	yes	na	low risk
Sallis et al. (1988)	yes	yes	yes	yes	yes	yes	yes	na	na	na	na	na	na	na	na	low risk
Brown et al. (1989)	yes	yes	yes	yes	yes	yes	yes	na	na	na	na	na	na	na	na	low risk
Anderssen and Wold (1992)	yes	yes	yes	yes	yes	yes	yes	na	na	na	na	na	na	na	na	low risk
Sallis et al. (1992)	yes	yes	yes	yes	yes	yes	yes	na	na	na	na	na	na	na	na	low risk
Rossow and Rise (1994)	yes	yes	yes	yes	yes	yes	yes	na	na	na	na	na	na	na	na	low risk
Biddle and Goudas (1996)	yes	yes	yes	yes	yes	yes	yes	na	na	na	na	na	na	na	na	low risk
Yang et al. (1996)	yes	yes	yes	no	yes	yes	yes	yes	no	na	na	na	na	na	na	having potential risk of bias
Yang et al. (1996)	yes	yes	yes	no	yes	yes	yes	yes	no	na	na	na	na	na	na	having potential risk of bias
Yang et al. (1996)	yes	yes	yes	no	yes	yes	yes	yes	no	na	na	na	na	na	na	having potential risk of bias
Trost et al. (1997)	yes	yes	yes	yes	yes	yes	yes	yes	yes	na	na	na	na	na	na	low risk
Vilhjalmsson and Thorlindsson (1998)	yes	yes	yes	yes	yes	yes	yes	na	na	na	na	na	na	na	na	low risk
Trost et al. (1999)	yes	yes	yes	yes	yes	yes	yes	na	na	na	na	na	na	na	na	low risk
McGuire et al. (2002)	yes	yes	yes	yes	yes	yes	yes	na	na	na	na	na	na	na	na	low risk

Davison et al. (2003)	yes	yes	yes	yes	yes	yes	yes	na	na	na	na	na	na	na	na	low risk
Ievers-Landis et al. (2003)	yes	yes	yes	yes	yes	yes	yes	na	na	na	na	na	na	na	na	low risk
Welk et al. (2003)	yes	yes	yes	yes	yes	yes	yes	na	na	na	na	na	na	na	na	low risk
Loucaides et al. (2004)	yes	yes	yes	yes	yes	yes	yes	na	na	na	na	na	na	na	na	low risk
Arredondo et al. (2006)	yes	yes	no	yes	yes	yes	no	na	na	na	na	na	na	na	na	having potential risk of bias
Davison et al. (2006)	yes	yes	yes	yes	yes	yes	yes	yes	yes	na	na	na	na	na	na	low risk
Ommundsen et al. (2006)	yes	yes	yes	yes	yes	yes	yes	na	na	na	na	na	na	na	na	low risk
Raudsepp (2006)	yes	yes	yes	yes	yes	yes	yes	na	na	na	na	na	na	na	na	low risk
DiNallo et al. (2007)	yes	yes	yes	yes	yes	yes	yes	na	na	na	na	na	na	na	na	low risk
Gattshall et al. (2008)	yes	yes	yes	yes	yes	yes	yes	na	na	na	na	na	na	na	na	low risk
Price et al. (2008)	yes	yes	yes	yes	yes	yes	yes	na	na	na	na	na	na	na	na	low risk
Sabiston and Crocker (2008)	yes	yes	yes	yes	yes	yes	yes	na	na	na	na	na	na	na	na	low risk
Anderson et al. (2009)	yes	yes	yes	yes	yes	yes	yes	na	na	na	na	na	na	na	na	low risk
Crimi et al. (2009)	no	no	no	no	yes	yes	yes	na	na	na	na	na	na	na	na	having potential risk of bias
Ha et al. (2009)	yes	yes	yes	yes	yes	yes	yes	na	na	na	na	na	na	na	na	low risk
Ha et al. (2009)	yes	yes	yes	yes	yes	yes	yes	na	na	na	na	na	na	na	na	low risk
Edwardson and Gorely (2010)	yes	yes	yes	yes	yes	yes	yes	na	na	na	na	na	na	na	na	low risk
Hardy et al. (2010)	yes	yes	yes	yes	yes	yes	yes	na	na	na	na	na	na	na	na	low risk
Heitzler et al. (2010)	yes	yes	yes	yes	yes	yes	yes	na	na	na	na	na	na	na	na	low risk
Kelly et al. (2010)	yes	yes	yes	yes	yes	yes	yes	na	na	na	na	na	na	na	na	low risk
Patnode et al. (2010)	yes	yes	yes	yes	yes	yes	yes	na	na	na	na	na	na	na	na	low risk
Fisher et al. (2011)	yes	yes	yes	yes	yes	yes	yes	na	na	na	na	na	na	na	na	low risk

Hendrie et al. (2011)	yes	no	no	yes	no	yes	yes	na	na	na	na	na	na	na	na	having potential risk of bias
Lee et al. (2011)	yes	yes	yes	yes	yes	yes	yes	na	na	na	na	na	na	na	na	low risk
Nikolaidis (2011)	no	yes	no	no	yes	yes	yes	na	na	na	na	na	na	na	na	having potential risk of bias
Rosenkranz and Dze-waltowski (2011)	yes	yes	yes	yes	yes	yes	yes	na	na	na	na	na	na	na	na	low risk
Wilson et al. (2011)	yes	yes	no	yes	no	yes	yes	yes	yes	yes	yes	no	yes	no	no	having potential risk of bias
Davison et al. (2012)	yes	yes	yes	yes	yes	yes	yes	na	na	na	na	na	na	na	na	low risk
Dumith et al. (2012)	yes	yes	yes	yes	yes	yes	yes	yes	yes	na	na	na	na	na	na	low risk
Lawman et al. (2012)	yes	yes	yes	yes	yes	yes	yes	na	na	na	na	na	na	na	na	low risk
McDavid et al. (2012)	no	no	no	yes	no	yes	yes	na	na	na	na	na	na	na	na	having potential risk of bias
Reimers et al. (2012)	no	yes	no	no	no	yes	yes	na	na	na	na	na	na	na	na	having potential risk of bias
Wilson and Spink (2012)	no	yes	yes	yes	no	yes	yes	no	yes	na	na	na	na	na	na	having potential risk of bias
Zhang et al. (2012)	no	yes	yes	no	no	yes	yes	na	na	na	na	na	na	na	na	having potential risk of bias
Crespo et al. (2013)	yes	yes	yes	yes	yes	yes	yes	na	na	na	na	na	na	na	na	low risk
Dewar et al. (2013)	yes	yes	no	yes	no	yes	yes	no	no	na	na	na	na	na	na	having potential risk of bias
Jackson et al. (2013)	no	yes	no	no	yes	yes	yes	na	na	na	na	na	na	na	na	having potential risk of bias
Leary et al. (2013)	no	yes	no	no	yes	yes	yes	na	na	na	na	na	na	na	na	having potential risk of bias
Loprinzi et al. (2013)	yes	no	no	yes	no	yes	no	na	na	na	na	na	na	na	na	having potential risk of bias
McMinn et al. (2013)	yes	yes	yes	yes	yes	yes	yes	na	na	na	na	na	na	na	na	low risk
Rutten et al. (2013)	yes	yes	yes	yes	yes	yes	yes	na	na	na	na	na	na	na	na	low risk
Chen et al. (2014)	yes	yes	yes	yes	yes	yes	yes	na	na	na	na	na	na	na	na	low risk

Farias Júnior et al. (2014)	yes	yes	yes	yes	yes	yes	no	na	na	na	na	na	na	na	na	having potential risk of bias
Gonzalez-Cutre et al. (2014)	no	yes	no	no	no	yes	yes	na	na	na	na	na	na	na	na	having potential risk of bias
Langer et al. (2014)	yes	yes	yes	yes	yes	yes	yes	na	na	na	na	na	na	na	na	low risk
Lawman and Wilson (2014)	yes	yes	yes	yes	yes	yes	yes	na	na	na	na	na	na	na	na	low risk
Lloyd et al. (2014)	yes	yes	yes	yes	yes	yes	yes	na	na	na	na	na	na	na	na	low risk
Määttä et al. (2014)	no	yes	yes	no	no	yes	yes	na	na	na	na	na	na	na	na	having potential risk of bias
(Silva et al., 2014)	no	yes	no	no	no	yes	yes	na	na	na	na	na	na	na	na	having potential risk of bias
Ling et al. (2015)	yes	yes	yes	yes	yes	yes	yes	na	na	na	na	na	na	na	na	low risk
Rhodes et al. (2015)	yes	yes	yes	no	no	yes	yes	no	yes	na	na	na	na	na	na	having potential risk of bias
Schoeppe and Trost (2015)	yes	yes	yes	yes	yes	yes	yes	na	na	na	na	na	na	na	na	low risk
Tate et al. (2015)	yes	yes	yes	yes	yes	yes	yes	na	na	na	na	na	na	na	na	low risk
Gontarev and Kalac (2016)	yes	yes	yes	yes	yes	yes	yes	na	na	na	na	na	na	na	na	low risk
Gontarev et al. (2016)	yes	yes	yes	yes	yes	yes	yes	na	na	na	na	na	na	na	na	low risk
Lee et al. (2016)	yes	yes	yes	yes	yes	yes	yes	na	na	na	na	na	na	na	na	low risk
McMurray et al. (2016)	yes	yes	yes	yes	yes	yes	yes	na	na	na	na	na	na	na	na	low risk
Silva et al. (2016)	yes	yes	yes	yes	yes	yes	yes	na	na	na	na	na	na	na	na	low risk
Van Kann et al. (2016)	yes	yes	yes	yes	yes	yes	yes	na	na	na	na	na	na	na	na	low risk
Verloigne et al. (2016)	no	yes	yes	no	no	no	yes	na	na	na	na	na	na	na	na	having potential risk of bias
Wing et al. (2016)	no	yes	yes	no	no	yes	yes	na	na	na	na	na	na	na	na	having potential risk of bias

Zarychta et al. (2016)	no	yes	no	yes	no	yes	yes	yes	no	na	na	na	na	na	na	having potential risk of bias
Egmose et al. (2017)	yes	yes	yes	yes	yes	yes	yes	yes	yes	na	na	na	na	na	na	low risk
Garriguet et al. (2017)	yes	yes	yes	yes	yes	yes	no	na	na	na	na	na	na	na	na	having potential risk of bias
Gillison et al. (2017)	yes	yes	yes	yes	yes	yes	yes	na	na	na	na	na	na	na	na	low risk
Larsen et al. (2017)	no	no	no	no	yes	yes	yes	na	na	na	na	na	na	na	na	having potential risk of bias
Liu et al. (2017)	yes	yes	yes	yes	yes	yes	yes	na	na	na	na	na	na	na	na	low risk
Quick et al. (2017)	yes	yes	yes	yes	yes	yes	yes	na	na	na	na	na	na	na	na	low risk
Tabak et al. (2017)	no	yes	no	no	yes	yes	yes	na	na	na	na	na	na	na	na	having potential risk of bias
Wang (2017)	no	yes	no	yes	no	yes	yes	na	na	na	na	na	na	na	na	having potential risk of bias
Wang et al. (2017)	yes	yes	yes	yes	yes	yes	yes	na	na	na	na	na	na	na	na	low risk
De la Torre-Cruz et al. (2018)	no	yes	no	no	yes	yes	yes	na	na	na	na	na	na	na	na	having potential risk of bias
Hamilton and Schwarzer (2018)	no	yes	yes	no	no	yes	yes	na	na	na	na	na	na	na	na	having potential risk of bias
Huffman et al. (2018)	yes	yes	yes	yes	yes	yes	yes	na	na	na	na	na	na	na	na	low risk
Liszewska et al. (2018)	no	yes	yes	no	no	yes	yes	no	yes	na	na	na	na	na	na	having potential risk of bias
Prioreschi et al. (2018)	yes	yes	yes	yes	yes	yes	yes	na	na	na	na	na	na	na	na	low risk
Robbins et al. (2018)	yes	yes	yes	yes	yes	yes	yes	na	na	na	na	na	na	na	na	low risk
Rodrigues et al. (2018)	yes	yes	yes	yes	yes	yes	yes	na	na	na	na	na	na	na	na	low risk
Sevil Serrano et al. (2018)	no	no	no	yes	yes	yes	yes	na	na	na	na	na	na	na	na	having potential risk of bias
Wilk et al. (2018)	yes	yes	yes	yes	yes	yes	yes	na	na	na	na	na	na	na	na	low risk

Biggs et al. (2019)	no	yes	no	yes	no	yes	yes	na	na	na	na	na	na	na	na	having potential risk of bias
Colabianchi et al. (2019)	yes	yes	yes	yes	yes	yes	yes	na	na	na	na	na	na	na	na	low risk
Costa et al. (2019)	no	yes	no	no	yes	no	yes	na	na	na	na	na	na	na	na	having potential risk of bias
Courtney et al. (2019)	no	no	yes	no	yes	no	yes	na	na	na	na	na	na	na	na	having potential risk of bias
George et al. (2019)	yes	yes	yes	no	yes	yes	yes	na	na	na	na	na	na	na	na	having potential risk of bias
Haidar et al. (2019)	no	yes	yes	no	no	no	yes	na	na	na	na	na	na	na	na	having potential risk of bias
Kobayashi et al. (2019)	yes	yes	yes	yes	yes	yes	yes	na	na	na	na	na	na	na	na	low risk
Lu et al. (2019)	yes	yes	yes	yes	yes	yes	yes	na	na	na	na	na	na	na	na	low risk
Mikalsen et al. (2019)	yes	yes	yes	yes	yes	yes	yes	na	na	na	na	na	na	na	na	low risk
Parker et al. (2019)	no	yes	no	yes	yes	no	yes	na	na	na	na	na	na	na	na	having potential risk of bias
Qurban et al. (2019)	no	yes	no	no	no	yes	yes	na	na	na	na	na	na	na	na	having potential risk of bias
Wang et al. (2019)	yes	yes	yes	yes	yes	yes	yes	na	na	na	na	na	na	na	na	low risk
Welch et al. (2019)	yes	yes	yes	yes	yes	yes	yes	na	na	na	na	na	na	na	na	low risk
Centeio et al. (2020)	yes	yes	yes	yes	yes	yes	yes	na	na	na	na	na	na	na	na	low risk
Lee and Lee (2020)	yes	yes	yes	yes	yes	yes	yes	na	na	na	na	na	na	na	na	low risk
Park and Park (2020)	yes	yes	yes	yes	yes	yes	yes	na	na	na	na	na	na	na	na	low risk
Ren et al. (2020)	yes	yes	yes	yes	yes	yes	yes	na	na	na	na	na	na	na	na	low risk
Sanz-Martín (2020)	yes	yes	yes	yes	yes	yes	yes	na	na	na	na	na	na	na	na	low risk
Zarychta et al. (2020)	no	yes	yes	yes	no	yes	yes	no	yes	na	na	na	na	na	na	having potential risk of bias
